# Letter to the Editor: Terminology around neurocognitive difference needs to change

**DOI:** 10.1186/s13063-026-09439-3

**Published:** 2026-01-15

**Authors:** Rebecca J. Linnett, Roseanne E. Billany, Agnes Fletcher, Matt Hammond, Noelle Robertson, Claire Warden, Polly-Anna Ashford

**Affiliations:** 1 Norwich Clinical Trials Unit, Norwich Medical School, Faculty of Medicine and Health Sciences, University of East Anglia, Norwich, NR4 7TJ UK; 2 Department of Cardiovascular Sciences and Partnership for Kidney Health Research, University of Leicester, Leicester, LE1 7RH UK; 3 Disability Rights UK, London, UK; 4 School of Psychology and Vision Sciences, University of Leicester, Leicester, LE1 7RH UK; 5 Sheffield Clinical Trials Research Unit, Sheffield, United Kingdom

Dear Editor,

We are a team of academics, clinicians, and experts by experience who are currently working on an NIHR-funded project to improve the accessibility of clinical trials for disabled people [1]. During the course of our research, we were concerned to note that the UK’s clinical study registry ISRCTN—which is, incidentally, managed by the same publisher as this journal—categorises neurocognitive differences such as autism, ADHD, dyslexia, and Tourette’s Syndrome under ‘Mental and Behavioural Disorders’.

Although these conditions are included in the Diagnostic and Statistical Manual of Mental Disorders, where they are characterised as ‘developmental disorders’ [2], we argue that this terminology is outdated and risks pathologising innate differences in a person’s neurotype that are intrinsic to the way they relate to the world rather than an inherent ‘disorder’ [3], focusing disproportionately on deficit rather than difference [4]. It also ignores the complexities associated with these diagnostic labels, such as the significant genetic and phenotypic heterogeneity amongst people diagnosed with any one of these ‘disorders’ [5], who each present with a unique profile of difficulties and strengths [6], and the frequent co-occurrence of these conditions [7]. Furthermore, characterising these neurocognitive differences as ‘disorders’ also ignores the significant strengths associated with them, including enhanced visual perception, strong spatial, auditory and semantic memory, superior empathy and higher levels of divergent thinking [6]. Indeed, we believe that using strength-based descriptors in registries, rather than focusing on ‘disorder’ or ‘impairment’, would go a long way towards helping to shift public and professional perceptions of people with neurocognitive differences.

We are also concerned that the category of ‘Mental and Behavioural Disorders’ is used by ISRCTN for studies relating to mental illness as well as those related to neurocognitive differences. Certainly, there is a greater risk of mental ill health for people with neurocognitive differences [e.g. [8, 9]]. However, these differences are not equivalent to psychiatric conditions and are often distinguished from the latter by their emergence in childhood and relative stability over the life course, as compared to mental illnesses, which are thought to generally have cycles of remission and relapse [7, 10]. We have noticed that ISRCTN routinely includes studies of neurocognitive differences in their ‘Mental and Behavioural Disorders’ category, even when the studies in question are not focused on mental illness or behaviour within these populations. For example, there are studies focused broadly on the physical health of autistic people [e.g. [11, 12]], people with ADHD [13], and people with Tourette’s Syndrome [14] that are categorised under ‘Mental and Behavioural Disorders’, whereas studies focused on the physical health of people with Down Syndrome or a learning disability are not [e.g. [15, 16]], despite these conditions also having mental and behavioural components.

We are aware that in many cases, researchers register studies themselves on ISRCTN and therefore are responsible for the condition category that the study is registered in. In other cases, records are populated by ISRCTN from NHS ethics applications on the Health Research Authority’s IRAS platform, and researchers are then asked to check the accuracy of the record before it is published. However, at present, researchers registering studies where the population has a neurocognitive difference are not given a viable alternative category to ‘Mental and Behavioural Disorders’ (or, in the case of IRAS, ‘Mental Illness’).

Our concern about this categorisation of neurocognitive differences is threefold. Firstly, many contemporary accounts of the experiences and narratives of people who come under this umbrella argue against pathologising terminology such as ‘disorder’ or ‘illness’ in favour of neurodiversity-affirming language [4, 17]. Secondly, most major funders of health research, at least within the UK, now insist on patient and public involvement (PPI) in the research that they are funding, meaning that ‘lay’ advisors and experts by experience are more involved in research than ever before. The continued use of outdated and pathologising language risks alienating these PPI advisors, putting up unnecessary barriers between the public and the medical community. Finally, the ‘Mental and Behavioural Disorders’ category is the largest on ISRCTN, with the vast majority of studies in this category being primarily about mental health. As well as there being ontological concerns about classifying neurocognitive differences in this way, the classification of studies about people with neurocognitive differences along with studies about mental illness also makes it practically very difficult when researchers are looking to identify studies about neurocognitive differences that are not also about mental health.

We are pleased to see that there is beginning to be a shift in some areas of the health research landscape around the terminology used for people with neurocognitive differences—for example, the NIHR, one of the primary funders of health research in the UK, explicitly encourages researchers to use inclusive language around neurocognitive difference in their journals rather than terms that focus on disorder or impairment [18]. We believe it is important that clinical trials registries such as the ISRCTN follow suit, and are therefore suggesting that the ‘Mental and Behavioural Disorders’ category on ISRCTN be retired in favour of two new categories: one to encompass studies relating to mental illness and mental health, and one to encompass studies of neurocognitive difference. Our suggestion is that the former is labelled ‘Mental Health’ and the latter is labelled ‘Neurocognitive Differences’ or ‘Neurodivergence’; this would mean that (e.g.) a study about depression in adults with ADHD would be assigned to both condition categories, but that otherwise studies of people with neurocognitive difference would be distinguished from studies about mental health. However, we appreciate that there will be varied opinions within the clinical and academic communities about the terminology that should be used, and we therefore see this letter as the beginning of a conversation about the categorisation of studies on ISRCTN and the Health Research Authority’s IRAS platform, as well as more broadly by researchers themselves.

## Data Availability

Not applicable.
